# The Role of γ-Tubulin in Centrosomal Microtubule Organization

**DOI:** 10.1371/journal.pone.0029795

**Published:** 2012-01-10

**Authors:** Eileen O'Toole, Garrett Greenan, Karen I. Lange, Martin Srayko, Thomas Müller-Reichert

**Affiliations:** 1 Boulder Laboratory for 3-D Electron Microscopy of Cells, University of Colorado Boulder, Boulder, Colorado, United States of America; 2 Max Planck Institute of Molecular Cell Biology and Genetics, Dresden, Germany; 3 Department of Biological Sciences, University of Alberta, Edmonton, Alberta, Canada; 4 Medical Theoretical Center, University of Technology Dresden, Dresden, Germany; Emory University, United States of America

## Abstract

As part of a multi-subunit ring complex, γ-tubulin has been shown to promote microtubule nucleation both *in vitro* and *in vivo*, and the structural properties of the complex suggest that it also seals the minus ends of the polymers with a conical cap. Cells depleted of γ-tubulin, however, still display many microtubules that participate in mitotic spindle assembly, suggesting that γ-tubulin is not absolutely required for microtubule nucleation *in vivo*, and raising questions about the function of the minus end cap. Here, we assessed the role of γ-tubulin in centrosomal microtubule organisation using three-dimensional reconstructions of γ-tubulin-depleted *C. elegans* embryos. We found that microtubule minus-end capping and the PCM component SPD-5 are both essential for the proper placement of microtubules in the centrosome. Our results further suggest that γ-tubulin and SPD-5 limit microtubule polymerization within the centrosome core, and we propose a model for how abnormal microtubule organization at the centrosome could indirectly affect centriole structure and daughter centriole replication.

## Introduction

In most animal cells, the centrosome is the major microtubule-organizing center (MTOC). It consists of a pair of centrioles surrounded by a complex, three-dimensional matrix of electron-dense, pericentriolar material (PCM) [1]. Observed over 100 years ago, centrosomes are typically 1.5–2 µm in diameter, but can vary depending on tissue type and cell size [1], [2]. Although many centrosomal components are now known [3], [4], [5], the composition and precise organization of the individual structural components within this complex organelle and the nucleation and outgrowth of microtubules from the centrosome are still not fully understood.

The PCM of the centrosome provides a kinetically favorable site for cellular microtubule nucleation as well as a structural hub for anchoring the microtubule minus ends [6]. Microtubule outgrowth results in a polarized microtubule astral array, with the majority of microtubules plus ends polymerizing away from centrosomes [7]. This arrangement of microtubules around centrosomes is exploited for a variety of functions including intracellular trafficking, cellular polarity, mitotic spindle assembly, and cytokinesis. The ability of a centrosome to promote the robust initiation of microtubule growth is due, in part, to γ-tubulin [8]. Two molecules of γ-tubulin and one copy each of the accessory proteins Spc97 and Spc98 compose the 300-kDa γ-tubulin small complex (γ-TuSC) [9]. In metazoans, multiple γ-TuSCs associate with additional proteins to form open γ-tubulin ring complexes (γTuRCs) [10], [11], which have been shown to serve as templates for polymerization of 13-protofilament microtubules [12], [13], [14], [15].

Despite the information on the mechanism of microtubule nucleation from γ-tubulin and associated proteins, the *in vivo* consequence of γ-tubulin depletion on the structure of the centrosomes and microtubules has not been addressed [16]. RNAi directed against the sole *C. elegans* γ-tubulin gene, *tbg-1*, results in a significant reduction but not complete loss of centrosomal microtubules [7], [17], [18], [19]. Defects also include aberrant spindle microtubule organization [18]. Studies using EB1::GFP to measure growing microtubule plus ends have shown that, although *tbg-1(RNAi)* embryos have ∼60% of wild-type centrosomal microtubule levels, the existing microtubules emanate from centrosomes with normal growth rate and polarity [7]. Therefore, the polarity of centrosomal microtubules in *tbg-1(RNAi)* embryos appear largely unaffected, which raises questions as to the function of γ-tubulin in microtubule arrangement and centrosome function. Although a decrease in centrosomal microtubule nucleation alone could interfere directly with mitotic microtubule-based processes, our data suggest that γ-tubulin plays an important role in organizing microtubule minus ends at the centrosome to create a polarized, radial microtubule array [20].

## Results

### γ-tubulin organizes capped microtubule ends at the centrosome periphery

**Figure 1:**
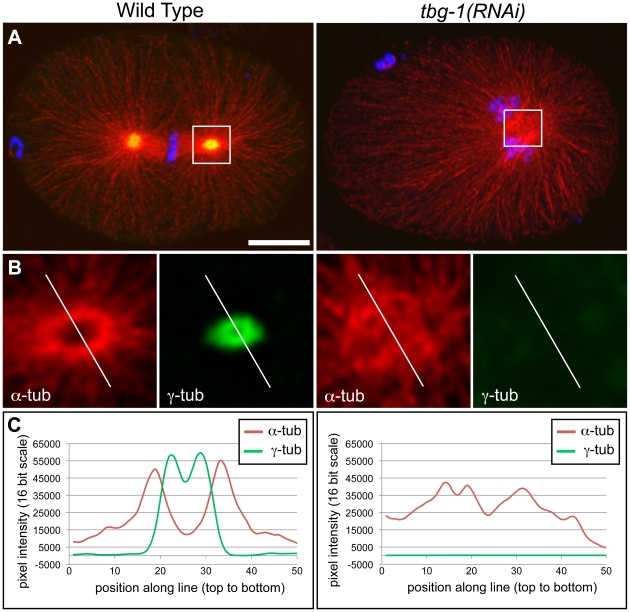
Distribution of α- and γ-tubulin in the *C. elegans* centrosome. A. Immunofluorescence images (projections of confocal stacks) of a wild-type embryo (left column) and a *tbg-1(RNAi)* embryo (right column) showing α-tubulin (red), γ-tubulin (green), and DNA (blue). B. Line scans from single confocal planes over centrosome regions as indicated in (A) used for measurements of pixel intensity. C. Linescan intensity plots of fluorescence intensity for α-tubulin (red) and γ-tubulin (green) along the marked lines presented in (B). In contrast to the *tbg-1(RNAi)* embryo, the concentration α-tubulin is locally reduced in the wild-type centrosome. Bar: 10 µm in A.

To investigate the role of γ-tubulin on mitotic centrosome organization, we first visualized the distribution of α-tubulin and γ-tubulin on immunostained wild-type and *tbg-1(RNAi)* embryos (Figure 1). A linescan through a wild-type centrosome revealed a clear reduction in α-tubulin levels within the core region. This core region coincided with a peak of fluorescence intensity of γ-tubulin (Figure 1A). In contrast, a linescan through the most concentrated mass of microtubules within a single confocal plane in the *tbg-1(RNAi)* embryos indicated that α-tubulin was not excluded in a similar pattern (Figure 1B–C). These results suggested that γ-tubulin provides a microtubule-organizing function for the centrosome that includes the formation of a microtubule-free zone within the central core, consistent with previous reports [18].

**Figure 2:**
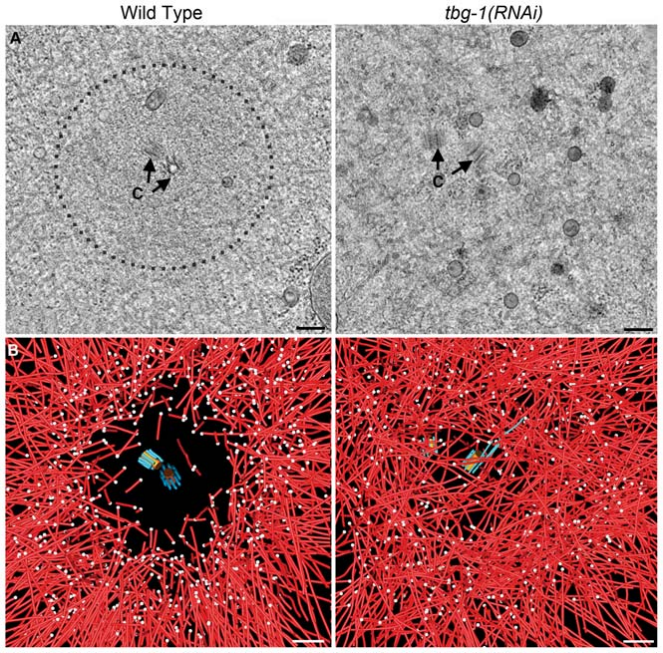
Structural organization of the mitotic centrosome in the one-cell wild-type and *tbg1(RNAi)* embryo. A. Tomographic slices of centrosomes containing centriole pairs (c) in one-celled metaphase embryos at early metaphase (wild-type: left column; *tbg-1(RNAi)*: right column) showing that the central core (dashed line) in the centrosome is lost after depletion of γ-tubulin. B. Modeling of centrioles (central tube in orange, centriolar microtubules in blue) and spindle microtubules (red) from the centrosome as shown in A. The microtubules in the *tbg-1(RNAi)* centrosome appear to traverse through and encircle the centrosome in contrast to the radial arrangement seen in wild-type embryos. The number of pole-proximal ends (white spheres) is reduced in the *tbg-1(RNAi)* embryo. Bars: 200 nm in A and B.

In order to elucidate the precise arrangement and structure of the microtubules nearest the centrosome, we performed electron tomography in combination with 3-D modeling on wild-type (Figure 2A) and *tbg-1(RNAi) C. elegans* embryos (Figure 2B). The centrosomes of wild-type, one-cell metaphase embryos appeared homogenous with a density characteristic of the PCM and an average diameter of ∼1500 nm (Figure 2A, dashed circle). Using tomography of 300 nm-thick serial slices, we modeled centrioles and all nearby microtubules to determine the position of their pole-proximal ends in 3-D (Figure 2A; Movie S1; Figure S1A for other examples). In agreement with fluorescence images and previous results [18], wild-type metaphase centrosomes displayed a “central core”, *i.e.* a zone of exclusion of pole-proximal microtubule ends around the centriole pair.

In contrast, the metaphase *tbg-1(RNAi)* embryo displayed vesicles within the centrosome core, suggesting a loss of centrosome integrity (Figure 2A). Furthermore, 3-D modeling revealed that numerous microtubules encircled or traversed the central region of the *tbg-1(RNAi)* centrosome, many coming within 100 nm of a centriole (Figure 2B; Movie S2; Figure S1B–C for other examples). In addition, fewer microtubule ends were observed in the centrosome of the *tbg-1(RNAi)* embryo compared to wild type (average of 497 *vs.* 970 from three *tbg-1(RNAi)* and two wild-type embryos, respectively; Table 1; Figure S1B–C for other examples).

**Table 1:** Comparison of wild type embryos with TBG-1 and SPD-5 depletion phenotypes.

Strain	# MT ends	Mean (± sd) distance MT ends from centrosome centre	# closed pole-proximal MT ends	# open pole-proximal MT ends	Mean distance closed ends centrosome centre	Mean distance open ends centrosome centre
**WT**						
(Fig. 1, 2)	866	766±192 nm	81%(702/866)	19%(164/866)	743±184 nm	867±195 nm
(Fig. S1A)	1076	725±201 nm	78%(835/1076)	22%(241/1076)	698±196 nm	818±191 nm
All WT	1942	743±255 nm	79%(1537/1942)	21%(405/1942)	719±192 nm	837±194 nm
***tbg-1(RNAi)***						
(Fig. 1, 2)	650	829±248 nm	30%(207/650)	64%(443/650)	786±241 nm	849±249 nm
(Fig. S1B)	415	661±235 nm	33%(138/415)	67%(277/415)	630±217 nm	676±242 nm
(Fig. S1C)	429	661±236 nm	26%(111/429)	74%(318/429)	608±219 nm	679±238 nm
All *tbg-1(RNAi)*	1494	734±255 nm	31%(456/1494)	69%(1038/1494)	695±243 nm	751±258 nm
***spd-5 (RNAi)***						
(Fig. 4E, F)	205	405±208 nm	46%(95/205)	54%(110/205)	332±200 nm	468±194 nm
(Fig. S2A)	284	395±200 nm	34%(98/284)	66%(186/284)	341±195 nm	424±198 nm
(Fig. S2B)	261	509±197 nm	36%(94/261)	64%(167/261)	414±147 nm	563±201 nm
(Fig. S2C)	652	558±235 nm	23%(147/652)	77%(505/652)	491±218 nm	578±237 nm
All *spd-5(RNAi)*	1402	494±229 nm	31%(434/1402)	69%(968/1402)	405±206 nm	533±228 nm

**Figure 3:**
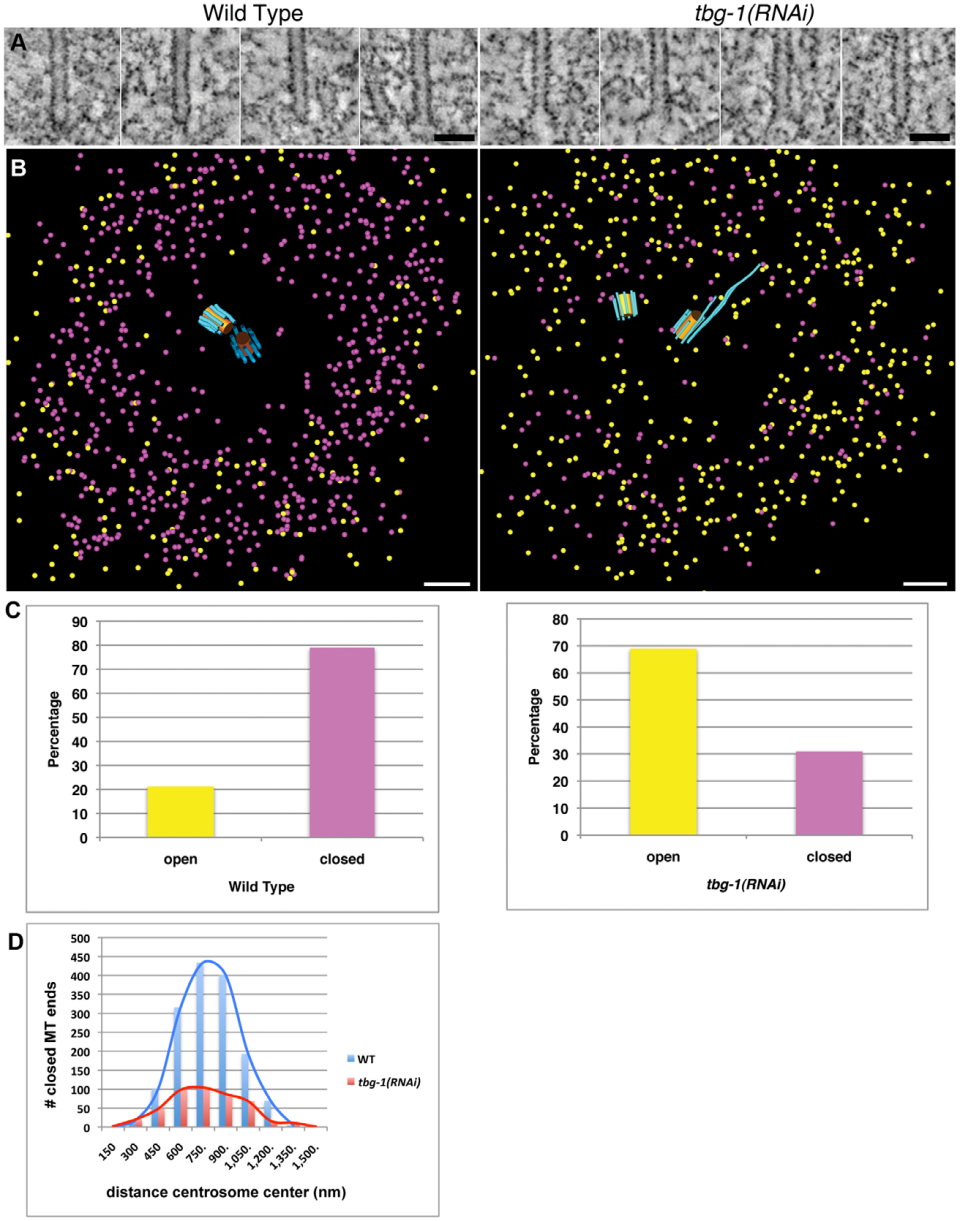
Distribution and morphology of pole-proximal microtubule ends in mitotic centrosomes. A. Gallery of pole-proximal ends (wild-type: left column; *tbg-1(RNAi)*: right column). Electron tomography reveals closed- and open-end morphologies. B. 3-D modeling of closed (purple spheres) and open (yellow spheres) microtubule ends. C. The ratio of closed/open pole-proximal ends is reversed in γ-tubulin compromised embryos. D. Distribution of closed microtubule pole-proximal ends. Although reduced in number, the closed pole-proximal ends in *tbg-1(RNAi)* embryos occupy a similar distance from the centrosome center as wild type. Bars: 50 nm in A; 200 nm in B.

Next, we examined the microtubule end morphology in the wild-type and *tbg-1(RNAi)* embryos. To image these ends in detail, we extracted a slice from the 3-D volume that contained the axis of each microtubule (Figure 3A). We identified closed (i.e. conical) and open (i.e. blunt or slightly flared) pole-proximal ends [21], and marked them with purple and yellow spheres, respectively (Figure 3B). In wild-type centrosomes, 79% (1537/1942; Table 1) of the ends nearest the centrioles were closed and 21% (405/1942) were open (Figure 3B). In contrast, only 30% (456/1494) of the pole-proximal ends were closed in *tbg-1(RNAi)* embryos and the rest (1038/1494) were open (Table 1 and Figure S1 for other examples). These results suggest that γ-tubulin contributes to the closed-end morphology of microtubules at the centrosome. We suspect that the residual capped ends in *tbg-1(RNAi)* result from incomplete depletion of γ-tubulin, however, the existence of a redundant microtubule end-capping mechanism cannot be ruled out. The presence of a few capped ends in *tbg-1(RNAi)* embryos allowed us to determine whether or not capped microtubules specifically maintain a proper position within the centrosome. The closed pole-proximal ends in *tbg-1(RNAi)* embryos occupied positions at an average of 695±243 nm from the center of the centrosome (Figure 3D; Table 1). These positions overlapped with their wild-type counterparts (average of 719±192 nm; Figure 1D; Table 1). This suggests that the end capping is necessary for proper microtubule positioning within the PCM.

### Depletion of γ-tubulin causes overly long centriolar microtubules

**Figure 4:**
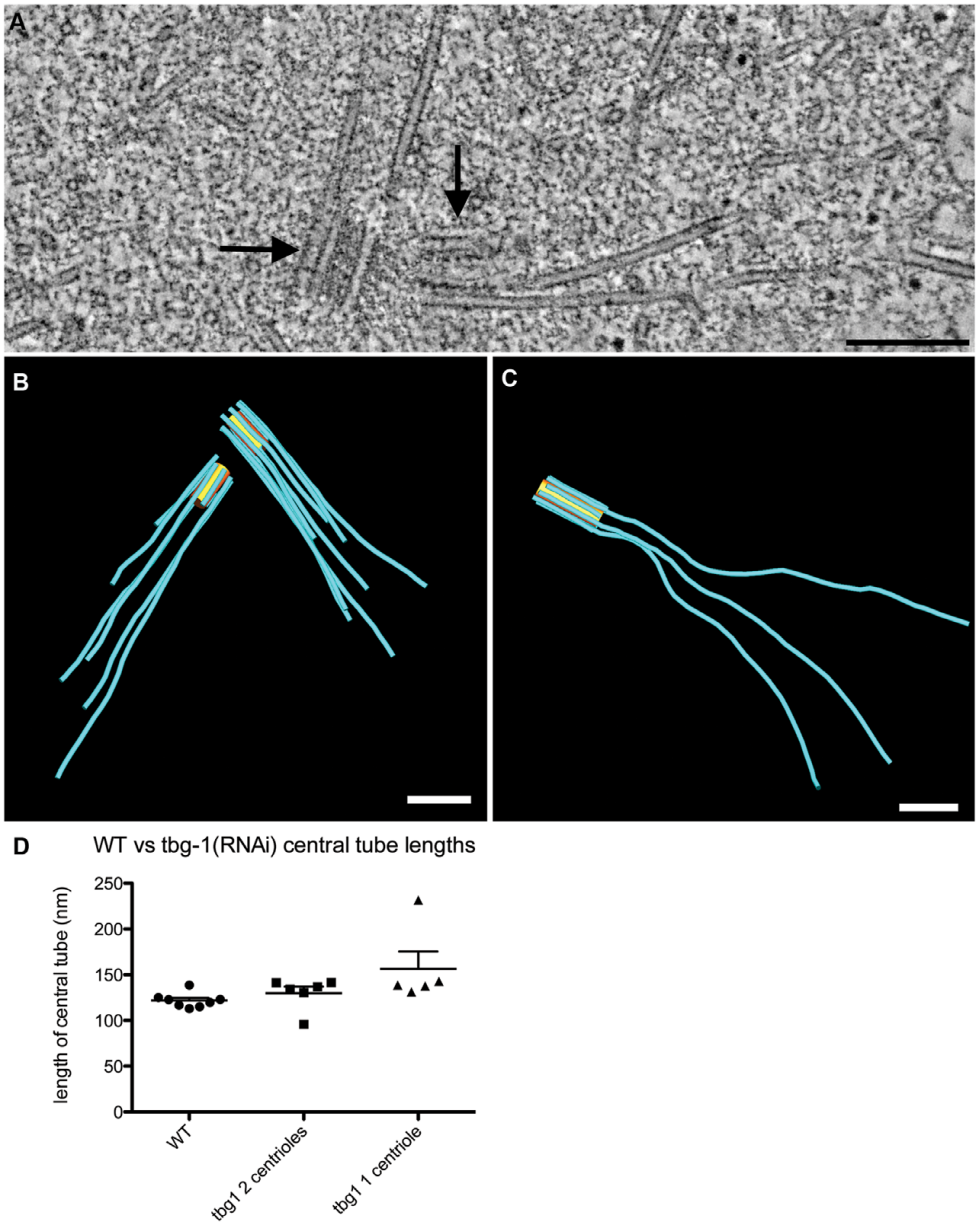
Effect of γ-tubulin-RNAi on centriole ultrastructure. A. Tomographic slice showing centriolar microtubules extending beyond the distal ends of the two centrioles (arrows). B–C. 3-D models of centrioles showing that both mother and daughter centrioles can contain elongated centriolar microtubules (B), however some tomograms contained only single centrioles (C). D. Length of central tubes from *tbg-1(RNAi)* centrosomes showing double or single centrioles. Bars: 200 nm A–C.

We next examined the ultrastructure of centrioles in *tbg-1(RNAi)* embryos. Wild-type cells contain two centrioles oriented at approximate right angles to one another. *C. elegans* centriole biogenesis initiates with the formation of a central tube that is subsequently surrounded by nine singlet microtubules. The singlet microtubules are normally not longer than the central tube at any stage during the first embryonic cell division [22]. Tomograms of *tbg-1(RNAi)* embryos revealed a consistent centriole defect whereby varying numbers of centriolar microtubules extended beyond the distal end of the central tube (Figure 4A–C; Movie S3; Figure S1 for more examples). This defect was observed on both the mother and the newly-assembled daughter centrioles. Most central tubes (10/11) had a mean length that was similar to wild type (Figure 3D), however, 1/11 centrioles had a central tube that was twice as long as wild type (Figure 4C). In some centrosomes (5/8), only one centriole was detected, or the daughter was incomplete. This suggests a role for TBG-1 in centriole formation, consistent with previous light microscopy experiments [23].

### Microtubule end placement in the centrosome periphery requires SPD-5

**Figure 5:**
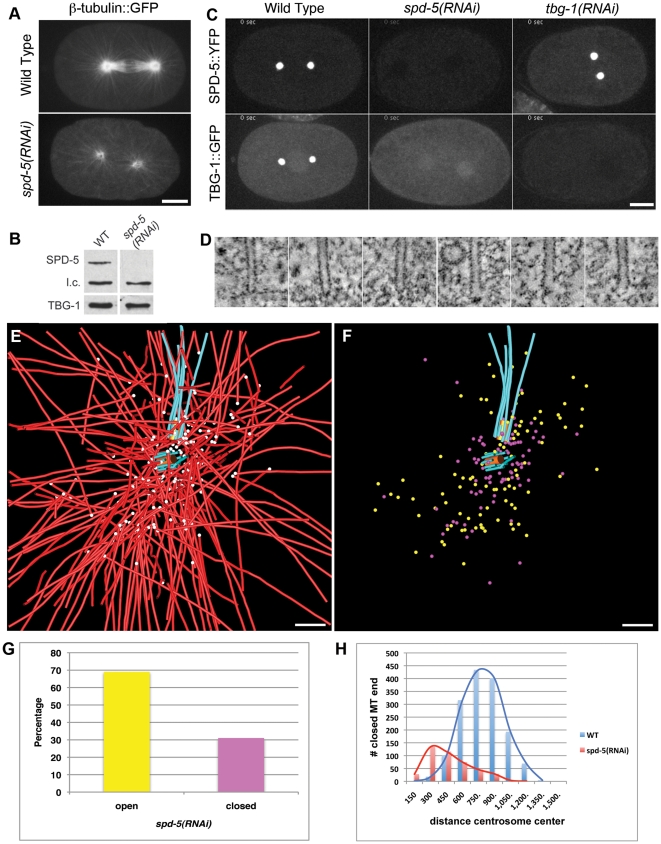
Effect of *spd-5(RNAi)* on centrosome structure and microtubule end morphology. A. Live-cell imaging of a wild-type and *spd-5(RNAi)* embryo expressing β-tubulin::GFP. B. Imaging of TBG-1::GFP in *spd-5(RNAi)* embryos. C. Western blot. γ-tubulin is present in the SPD-5-compromised embryo. A non-specific band from the anti-SPD-5 antiserum served as a loading control (l.c.). D. Gallery of closed (left images) and open (right images) pole-proximal ends in *spd-5(RNAi)* embryos. E. Modeling of centrioles (central tube in orange, centriolar microtubules in blue) and spindle microtubules. Compared to wild type, the number of pole-proximal ends (white spheres) is reduced and these ends are distributed near the centrioles within the centrosome core. The centriolar microtubules are extended. F. 3-D modeling of closed (purple spheres) and open (yellow spheres) microtubule ends. G. The closed microtubule pole-proximal ends are closer to the centrosome center compared to wild type. H. Percentage of closed *vs.* open pole proximal ends. Bars: 10 mm A and C; 50 nm D; 200 nm E and F.

**Figure 6:**
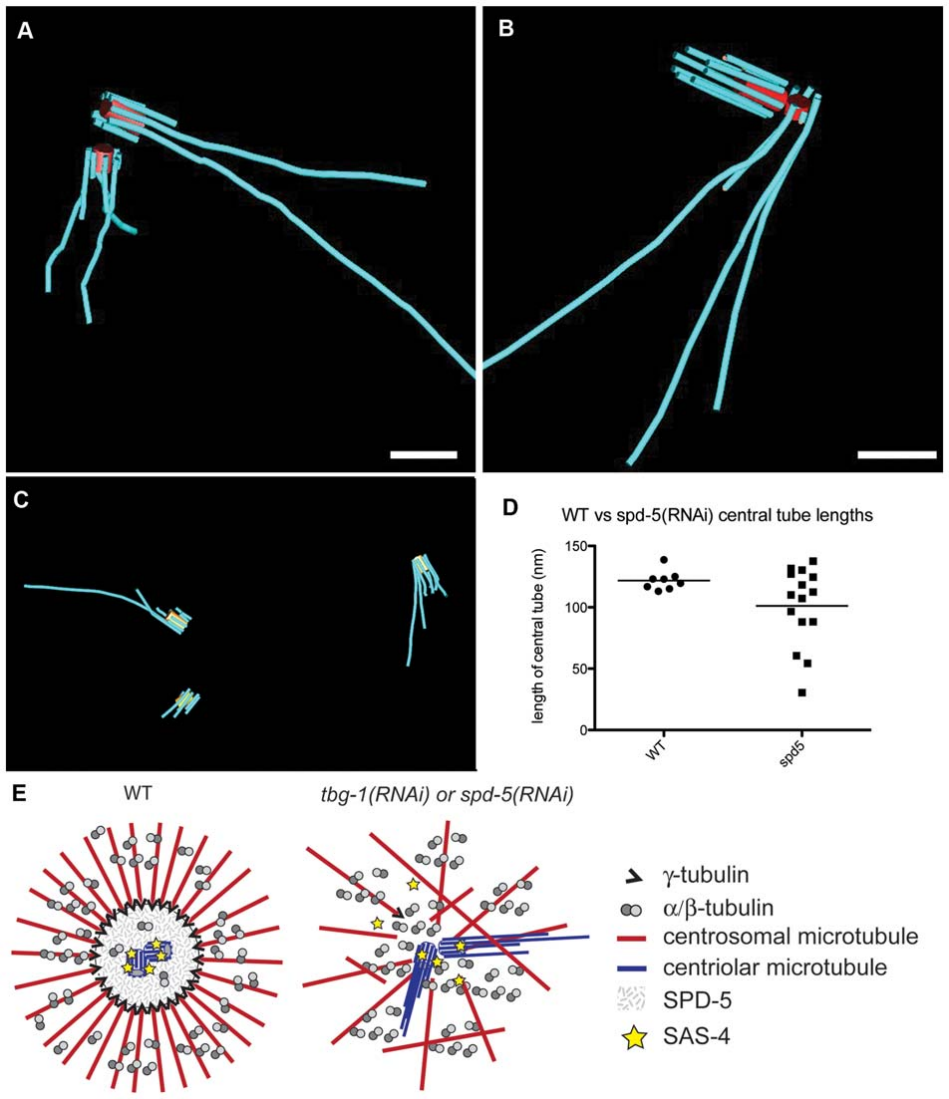
Model explaining the relationship between centrosomal microtubule organization and centriole morphology. A–B. Centriole pairs in *spd-5(RNAi)* embryos with variable lengths of the central tube of daughter centrioles. C. Centrosome region with three centrioles at one pole. Although it is unclear how one extra centriole could arise within a single cell cycle, the loss of SPD-5 may cause excessive movement of mother and daughter centrioles during the duplication process. If a daughter is dislodged at an early stage of this process, perhaps the same mother centriole can support the assembly of a second daughter. D. Quantification of central tube length in wild-type versus *spd-5(RNAi)* embryos. E. Model explaining the effect of TBG-1 and SPD-5 depletion on the structural organization of the centrosome. In wild type, the PCM component SPD-5 and γ-tubulin might contribute to a zone of exclusion visible as a ring-shaped area of pole-proximal microtubule ends around centrioles that limits microtubule polymerization and physically prevents movement of microtubule polymers into the centrosome core. Centriolar microtubule assembly occurs normally during late prophase, in a SAS-4 dependent manner. The zone of exclusion might trap SAS-4 within this central core, which could specifically promote the assembly of centriolar microtubules in an environment expected to be otherwise non-conducive to microtubule polymerization. In *tbg-1(RNAi*) or *spd-5(RNAi)* embryos, microtubule polymerization occurs near the centrioles, exchange with cytoplasmic SAS-4 becomes possible, and centriolar microtubules over-extend. Microtubule numbers are greatly reduced in *spd-5(RNAi)* but less affected in *tbg-1(RNAi)*, where the majority of microtubule ends near the centrosome exhibit an open, rather than a closed morphology. Bars: 200 nm A–C.

We further tested whether the centriolar microtubule defects were due directly to the loss of γ-tubulin or an indirect consequence of PCM dysfunction and subsequent disorganization of the microtubule array. SPD-5 is a major PCM component in *C. elegans*; it is required for centrosome maturation via the recruitment of γ-tubulin and other core components [24], [25], [26] and is essential for robust microtubule outgrowth (Figure 5A; Movie S4) [24]. We used *spd-5(RNAi)* to compromise centrosome structure/function and interfere with the intracellular distribution of TBG-1 (Figure 5B–C). We applied electron tomography to visualize the distribution and end morphology of the microtubules (Figure 5D). Similar to *tbg-1(RNAi)* embryos, microtubules in *spd-5(RNAi)* traversed the centriole vicinity (Figure 5E–F; Movie S5; and Figure S2 for other examples). We determined the ratio of closed-to-open pole-proximal ends within this 3-D model. The majority of the microtubule pole-proximal ends were open (Figure 5G; Table 1). Microtubules in the *spd-5(RNAi)* embryos might originate from the chromatin rather than centriolar regions, therefore it is possible that many of these open ends could represent growing plus tips [7], [24], [27]. Interestingly, unlike the *tbg-1(RNAi)* embryos, where the few capped ends that remained were situated at near-wild-type distances from the centrosome center, the capped ends in *spd-5(RNAi)* embryos were very close to the centrosome center (405±206 nm; Table 1). Therefore both SPD-5 and TBG-1 are important for the proper positioning of microtubule ends around the centrioles. In addition, the centrioles in the *spd-5(RNAi)* embryos had structural defects similar to *tbg-1(RNAi)* embryos, with distal centriolar microtubule ends extending beyond the central tube (Figure 6A–C; and Figure S2). However, the daughter central tubes were shorter in *spd-5(RNAi)* embryos than in wild type (Figure 6D).

## Discussion

Taken together, our observations suggest that γ-tubulin is important for the proper microtubule end-morphology and end-placement in the periphery of the centrosome (Figure 6E). In other systems, pericentrin, a large coiled-coil-containing protein, has been implicated in anchoring microtubule minus ends to the PCM [28]. In *C. elegans*, SPD-5 is required for centrosome structure and recruitment of TBG-1 and other factors [24]. Our results further suggest that microtubule-end placement in the centrosome periphery also requires SPD-5. The placement of microtubules into the PCM could also have consequences for microtubule polymer dynamics, as many regulators of microtubules localize to centrosomes in a γ-tubulin-dependent manner [6], [29], [30]. In the absence of functional γ-tubulin, microtubules in *Drosophila* S2 cells assemble near the centrosome, but they become unusually long and curved [31], consistent with our findings in *C. elegans tbg-1(RNAi)* embryos.

When nucleated from γ-tubulin *in vitro*, microtubules minus ends display a closed, cone-shaped morphology [14], [32], [33]. Such cone-shaped ends are reminiscent of microtubule minus ends observed at yeast spindle pole bodies [34] and at isolated *Drosophila* centrosomes [35]. In wild-type nematode embryos, the closed, centrosome-proximal microtubule ends outnumber the open ends 4∶1, and the majority of the open ends are on kinetochore microtubules [21]. Our electron tomography results verify that γ-tubulin contributes a structural role to microtubule ends *in vivo*. The frequency of the conical cap was also greatly diminished in *spd-5(RNAi)*, and we suggest two likely explanations for this result. First, because of the severe reduction in centrosomal microtubule nucleation in *spd-5(RNAi)* [23], [24], many of the open ends we observed near the centrioles could be plus-ends originating from chromatin-initiated microtubules as suggested by others [7], [24], [27]. Second, the PCM scaffold in wild-type cells might facilitate microtubule minus-end capping by recruiting γ-tubulin and providing a docking site for the γ-tubulin ring complex [6].

The over-extension of centriolar microtubules in *tbg-1(RNAi)* embryos seems inconsistent with the established role for γ-tubulin in promoting microtubule polymerization *in vitro*. However, centriolar microtubule extension has also been observed in γ-tubulin-depleted *Drosophila* S2 cells [31] and in cells depleted of the distal centriole microtubule-capping protein CP110 [36], [37], [38]. This raises the possibility that γ-tubulin could play additional roles in recruiting and/or activating this type of protein [6], although a CP110p equivalent is not apparent in *C. elegans*.

To date, SAS-4 is the only centriolar component required specifically for the formation of the centriolar microtubules and not the central tube [22], [39]. Furthermore, mammalian tissue culture cells over-expressing the SAS-4 homologue, CPAP, have over-extended centriolar microtubules. Interestingly, in a previous *C. elegans* study using light microscopy and photobleaching techniques, γ-tubulin was shown to be important for the stabilization of a pool of SAS-4 protein at the centrioles during later stages of centrosome maturation [40]. It was speculated that loss of SAS-4 stability at the centrosome in *tbg-1(RNAi)* embryos might directly interfere with centriolar microtubule assembly. We observed single centrioles in over half of the *tbg-1(RNAi)* embryos, indicating a role for γ-tubulin in daughter centriole formation. However, the over-extension of centriolar microtubules in *tbg-1(RNAi)* embryos also suggests that γ-tubulin is not required for centriolar microtubule production but may be important in regulating the extent of the process.

*In tbg-1(RNAi)* embryos, mis-positioning of microtubule ends at the centrosomes could also contribute to a deregulation of tubulin polymer dynamics within the centrosome core. Normally, the arrangement of all capped microtubule minus ends facing inward would be expected to limit microtubule polymerization in the central region due to low free tubulin concentration. Observations of a centrosomal core region have been reported as early as 1897 by E. B. Wilson [41]. In our study, we found a clear reduction in the level of α-tubulin within the wild-type centrosome core by immunofluorescence and an absence of microtubules in this area via EM tomography. Our results further suggest that both γ-tubulin and SPD-5 contribute to the formation of the microtubule-free zone around the centrioles. We therefore speculate that γ-tubulin could facilitate the formation of a microtubule-free zone in at least two ways. First, a high concentration of γ-tubulin in the centrosome could physically restrict the movement of free α/β tubulin subunits into the core, thus limiting polymerization of microtubules. Second, γ-tubulin could limit microtubule polymerization within the core because of its own polarized placement in the centrosome, such that any free α/β tubulin would be expected to polymerize away from the center exclusively.

An inappropriate increase in overall microtubule polymerization within the centrosome core would also explain why the centriolar extensions in *tbg-1(RNAi)* embryos are not limited to the newly forming centriole but are also apparent on the sperm-derived mother centrioles. Furthermore, pushing forces resulting from polymerization of microtubules near to and between centrioles could also explain why the centrioles are often separated from one another in *tbg-1(RNAi)* and *spd-5(RNAi)* embryos.

It has been reported that γ-tubulin is required to immobilize SAS-4 within mature centrosomes [40]. Early SAS-4 recruitment to centrioles could occur prior to the formation of this zone of exclusion, but once established as the centrosome matures, a certain fraction of SAS-4 could become trapped within the central core. The SAS-4 homologue, CPAP, promotes centriolar microtubule assembly through binding of tubulin heterodimers to promote procentriole elongation [36], [37], [38], [42]. We suggest that *C. elegans* SAS-4 might be necessary for controlled centriolar microtubule growth within a centrosomal core that is otherwise non-conducive to microtubule polymerization. A centrosomal core environment that limits microtubule polymerization could be important to maintain the structural integrity of centrosomes, ensuring that mother and daughter centrioles do not separate prematurely, due to physical forces arising from inappropriate microtubule assembly.

## Materials and Methods

### RNAi Methods, Light Microscopy and Quantification of Fluorescence

Wild-type or TH27 [β-tubulin::GFP; Histone::GFP] worms were injected with dsRNA as in Sönnichsen *et al.* [4] and cultured at 25°C for 22–28 hr prior to dissection. Loss-of-function phenotypes were first confirmed by observing the RNAi embryos via fluorescence or light microscopy prior to freezing. Confocal fluorescence images of fixed and living embryos were obtained with a Hamamatsu Orca R2 camera on an inverted Olympus IX81 (60×, NA 1.42 oil objective) microscope with a Yokogawa CSU-10 spinning disc confocal head (Quorum Technologies) controlled by MetaMorph software. Embryos were fixed for immunofluorescence as previously described [43]. Wild-type and *tbg-1(RNAi)* embryos were combined and fixed on the same slide and treated with primary antibodies DM1A (10 µg/mL, mouse monoclonal, Sigma) and rabbit-anti-TBG-1 (35 µg/mL) [19] and fluorescently-labeled secondary antibodies and Alexa 488 anti-mouse and Alexa 546 anti-rabbit secondary antibodies (Invitrogen). DAPI (1 µg/mL) was used to visualize the chromatin. Image stacks were deconvolved using Huygens Essential software and maximum projections of image stacks were generated for the whole embryo using false colors for display. Linescans (50 pixels long) were performed on a single deconvolved image plane exhibiting the highest intensity of α-tubulin within a circle (10 µm diameter) using Metamorph software. Orientation of the line was arbitrary and did not alter the overall conclusions.

### Immunoblotting

L4 larvae were injected with *spd-5(RNAi)*. After 27–30 h at 20°C, 50 wild-type and *spd-5(RNAi)* worms were transferred to 1 mL of M9 buffer and embryos were isolated as previously described [19]. Samples were run on a 10% SDS-PAGE gel and immunoblots were bisected at 70 kDa. Immunoblots were then probed using rabbit anti-SPD-5 or rabbit anti-γ-tubulin. Binding of SPD-5 (135 kDa) and TBG-1 (50 kDa) were detected with an HRP-conjugated secondary antibody (1∶5000; Bio-Rad Laboratories).

### Specimen Preparation for Electron Microscopy

*C. elegans* hermaphrodites were dissected in M9 buffer containing 20% BSA (Sigma). Single embryos were collected into capillary tubing to observe the development of the one-celled embryos as described [44]. Embryos at specific stages were transferred to 100 µ-deep membrane carriers (Leica) and frozen using the EMPACT2+RTS high-pressure freezer (Leica). Specimens were then freeze-substituted in anhydrous acetone containing 1% osmium tetroxide and 0.1% uranyl acetate over 24–48 h. Epon/Araldite infiltrated samples were flat embedded in a thin layer of resin and polymerized for 2–3 d at 60°C. Serial semi-thick sections (300 nm) were cut using a Leica UCT ultramicrotome. Sections were collected on Formvar-coated copper slot grids and post-stained with 2% uranyl acetate in 70% methanol and Reynold's lead citrate.

### Electron Tomography

Dual-axis electron tomography was performed as described [44]. Briefly, 15-nm colloidal gold particles (Sigma-Aldrich) were attached to each surface of the semi-thick sections to serve as fiducial markers for subsequent image alignment. Tilt series datasets were imaged using a TECNAI F30 intermediate-voltage electron microscope (FEI) operated at 300 kV. The SerialEM program [45], was used to automatically acquire images every 1° over a ±60° range using a Gatan 2K×2K CCD camera at a pixel size of 1.2 nm. Tomograms from 3–4 serial sections were calculated using the IMOD software package and joined to produce a final volume containing most of the centrosome (2.4 µm×2.4 µm×0.9 µm) [46], [47]. We recorded 4 wild-type centrosomes, 8 *tbg-1(RNAi)* centrosomes, and 7 centrosomes of *spd-5(RNAi)* embryos. By electron tomography we checked that GFP-tubulin labeling had no effect on the morphology of the pole-proximal microtubule ends.

### Modeling and Analysis of Tomographic Data

Tomograms were displayed and analyzed using the IMOD program, *3dmod* [46]. The centrioles, including the central tube (orange) and the centriolar microtubules (light blue) were modeled in 8 wild-type, 11 *tbg-1(RNAi)*, and *15 spd-5(RNAi)* centrioles. The centrosomal microtubules (red) as well as closed (purple spheres) and open (yellow spheres) microtubule pole-proximal ends were modeled and tracked throughout the volume of 2 wild-type, 3 *tbg-1(RNAi)* and 4 *spd-5(RNAi)* centrosomes. In addition, we modeled closed in the centrosome. The projections of the 3-D models were displayed and rotated to study their 3-D geometry. To display in 3-D, substructures, such as microtubules, were meshed using the IMODMESH program and shown as tubular graphical objects. Measurements of centrosome components were extracted from model contour data using the companion program IMODINFO. The 3-D distance of microtubule pole-proximal ends to a reference point in the centrosome center was calculated using the program IMOD-DIST.

## Supporting Information

Figure S1 **Three-dimensional modeling of centrosome regions in early** ***C. elegans*** **embryos.** A. Wild-type embryo. B–C. γ-tubulin compromised embryos. The distribution of closed (purple) and open (yellow) microtubule ends and centrioles is shown on the right. Bars: 200 nm in A–C.
(TIFF)

Figure S2 **Three-dimensional modeling of** ***spd-5(RNAi)*** **embryos.** The distribution of closed (purple) and open (yellow) microtubule ends and centrioles is shown on the right. Bars: 200 nm in A–C.
(TIFF)

Movie S1 **Model of a reconstructed centrosome region of a wild-type embryo (corresponding to** **Figure 2B** **, left).**
(MOV)

Movie S2 **Model of a reconstructed centrosome region of a** ***tbg-1(RNAi)*** **embryo (corresponding to** **Figure 2B** **, right).**
(MOV)

Movie S3 **Model of a reconstructed** ***tbg-1(RNAi)*** **centriole pair (corresponding to** **Figure 4A** **).** The movie shows the central tubes and the centriolar microtubule extensions.
(MOV)

Movie S4 **Live-cell imaging of a wild-type (left) and** ***spd-5(RNAi)*** **embryo (right) expressing β-tubulin::GFP (corresponding to** **Figure 5A** **).**
(MOV)

Movie S5 **Model of a reconstructed centrosome region of a** ***spd-5(RNAi)*** **embryo (corresponding to** **Figure 6E** **).**
(MOV)
