# Correction: The Role of γ-Tubulin in Centrosomal Microtubule Organization

**DOI:** 10.1371/annotation/c303c4e9-edae-4602-96b6-21c3796b89c9

**Published:** 2012-02-23

**Authors:** Eileen O'Toole, Garrett Greenan, Karen I. Lange, Martin Srayko, Thomas Müller-Reichert

The funding statement is incomplete. The complete statement is: "This work was supported by an operating grant from the Natural Sciences and Engineering Research Council of Canada as well as scholar awards from Alberta Heritage Foundation for Medical Research and the Canadian Institutes of Health Research to MS and grants from the Deutsche Forschungsgemeinschaft (DFG: MU 1423/2-1 and MU 1423/3-1) and a Human Frontier Science Program grant (RGP 0034/2010) to TMR. Tomography in the Boulder Laboratory for 3D EM of cells was supported by grant RR-000592 from the National Center for Research Resources of the National Institutes of Health to Andreas Hoenger.The funders had no role in study design, data collection and analysis, decision to publish, or preparation of the manuscript."

