# Supplementary figures and images for: The Role of γ-Tubulin in Centrosomal Microtubule Organization
Source: PLoS One. 2012 Jan 10;7(1):e29795. doi: 10.1371/journal.pone.0029795 (PMC3254605; doi:10.1371/journal.pone.0029795)

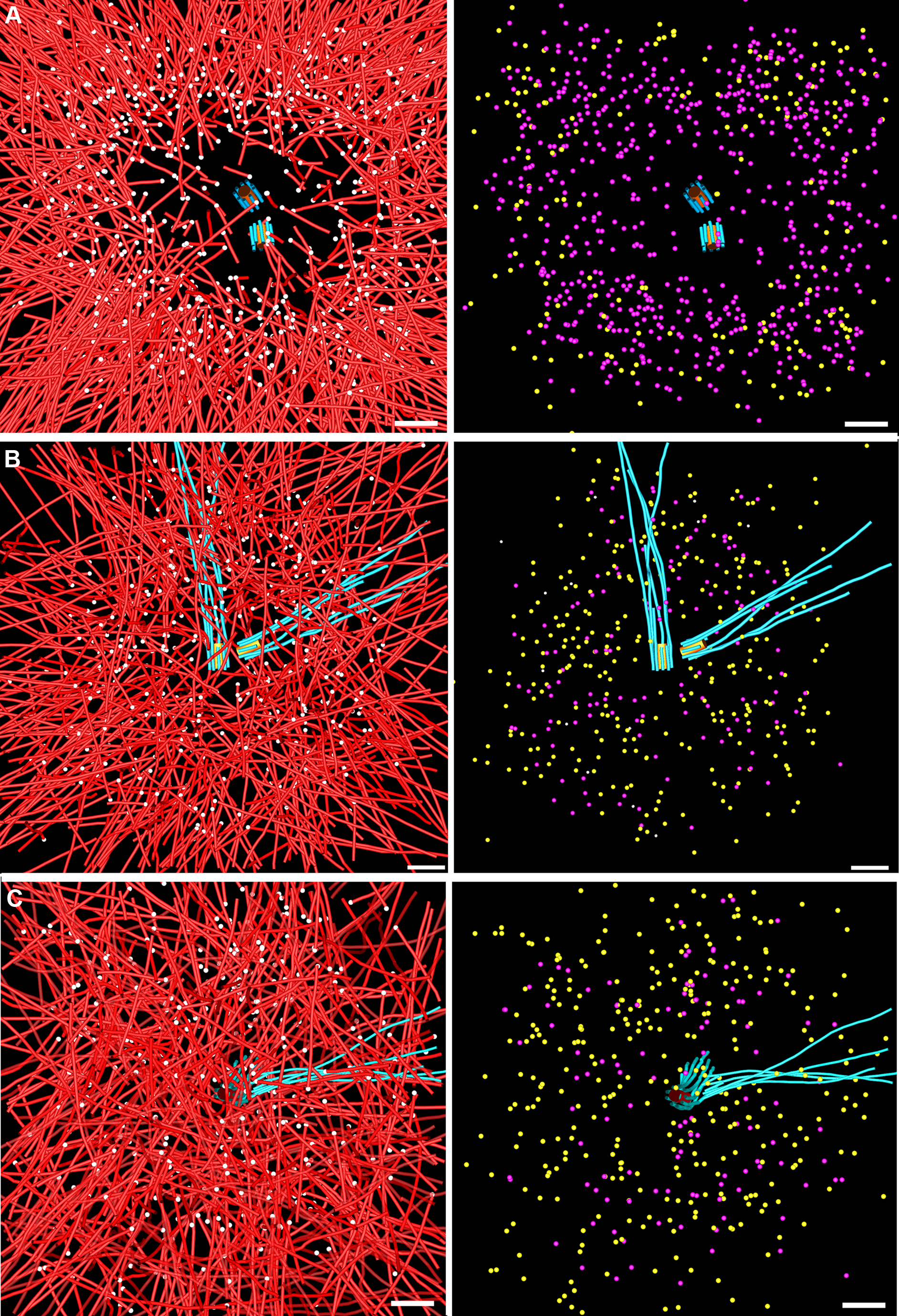

Supplement: Figure S1 — Three-dimensional modeling of centrosome regions in early C. elegans embryos. A. Wild-type embryo. B–C. γ-tubulin compromised embryos. The distribution of closed (purple) and open (yellow) microtubule ends and centrioles is shown on the right. Bars: 200 nm in A–C. (TIFF) [file pone.0029795.s001.tiff]

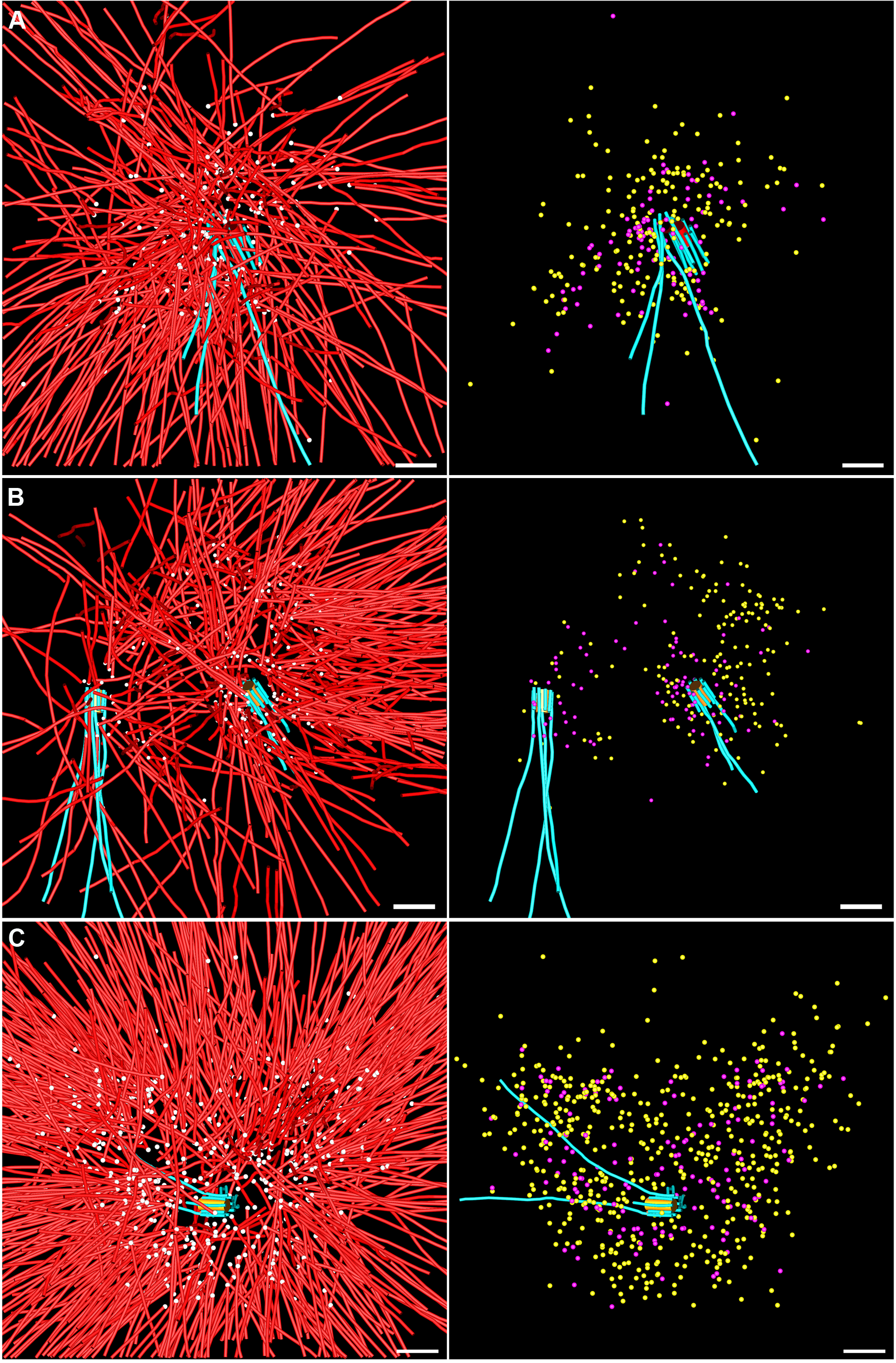

Supplement: Figure S2 — Three-dimensional modeling of spd-5(RNAi) embryos. The distribution of closed (purple) and open (yellow) microtubule ends and centrioles is shown on the right. Bars: 200 nm in A–C. (TIFF) [file pone.0029795.s002.tiff]
